# Pacmanvirus S19, the Second Pacmanvirus Isolated from Sewage Waters in Oran, Algeria

**DOI:** 10.1128/MRA.00693-21

**Published:** 2021-10-21

**Authors:** Khalil Geballa-Koukoulas, Souhila Abdi, Bernard La Scola, Guillaume Blanc, Julien Andreani

**Affiliations:** a MEPHI, APHM, IRD 198, Aix Marseille Université, IHU-Méditerranée Infection, Marseille, France; b Aix Marseille Université, Université de Toulon, CNRS, IRD, MIO UM 110, Marseille, France; Portland State University

## Abstract

Acanthamoeba castellanii is an amoeba host that was used to isolate a novel strain named pacmanvirus S19. This isolate is the second strain reported and belongs to the extended *Asfarviridae* family. Pacmanvirus S19 harbors a 418,588-bp genome, with a GC content of 33.20%, which encodes 444 predicted proteins and a single Ile-tRNA.

## ANNOUNCEMENT

In 2017, the first pacmanvirus (strain A23) was isolated from Acanthamoeba castellanii (1) and nested phylogenetically within the extended *Asfarviridae* clade (2). This clade contains the African swine fever virus, a virus with an endemic background that causes swine disease and death (3–7). Faustoviruses (8), kaumoebaviruses (9), abalone asfarvirus-like virus (10), and asfarvirus metagenome-assembled genomes (MAGs) (11) complete the known diversity of this clade. Here, we report the genome sequence of pacmanvirus S19, which was isolated from a sewage sample collected in Cap Falcon, Oran, Algeria (35°46′15.3″N, 0°47′47.2″W), and stored at 4°C before analysis. This virus was isolated using a coculture technique on a 24-well plate, as described by Andreani et al. (1); viral DNA was extracted with an EZ1 Advanced XL automated system (Qiagen, France). A 2 × 251-bp paired-end sequencing strategy was used, and limited-cycle PCR amplification (12 cycles) completed the tag adapters and introduced dual-index barcodes. After purification on beads, the library was normalized according to the Nextera XT protocol (Illumina) before sequencing on an Illumina MiSeq instrument (8) in a 39-hour single run.

**FIG 1:**
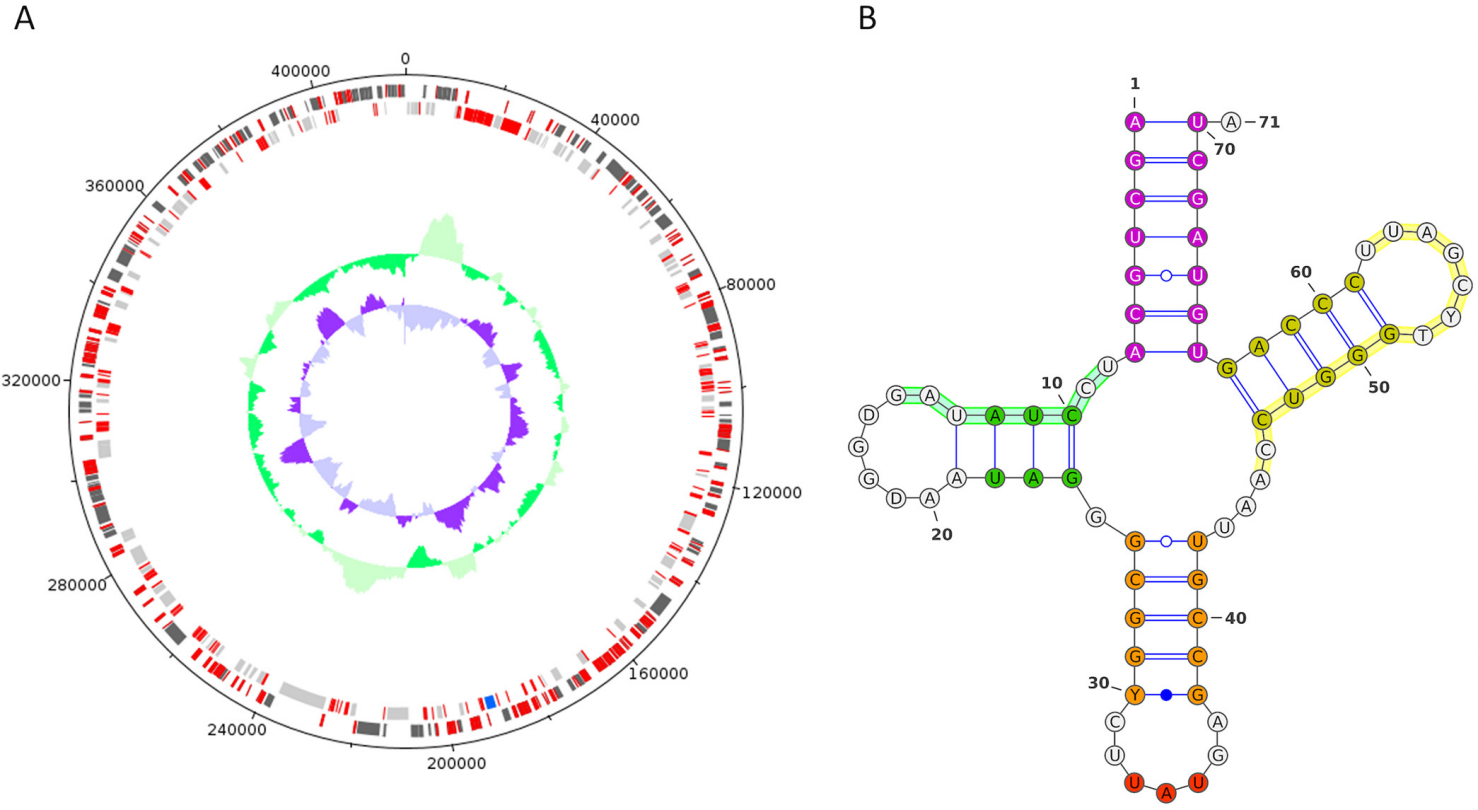
Circular representation of the pacmanvirus S19 genome with its tRNA. (A) The major capsid protein (MCP) gene is marked with a blue rectangle. Hypothetical proteins are highlighted in red. The outer ring corresponds to the positive strand of the genome, while the inner ring corresponds to the negative strand. GC skew is indicated by the small inner purple plot in the center of the genome and the GC content by the outer green plot. Construction of the plot was performed via DNAPlotter (22). (B) The Ile anticodon is marked in red. Orange represents the anticodon stem. Purple corresponds to the amino-acyl stem, while green represents the D loop stem and yellow represents the TPsiC stem. D loop and TPsiC signals are highlighted with light green and yellow lines, respectively. For the construction of tRNA, the predicted sequence was uploaded to the tRNAmod online tool (23). Information about the tRNA annotation was obtained using ARAGORN (19). The final representation was prepared using VARNA (24).

Sequencing yielded 2,586,744 raw reads, which were trimmed and quality controlled by AlienTrimmer (12) (with parameters p = 80, l = 100, and k = 10) before *de novo* assembly with SPAdes v 3.11.1 (13) with k-mer sizes of 21, 55, 77, 99, and 127. Remaining gaps were closed by subassembly of reads aligned with HISAT (14) onto orthologous A23 genomic regions corresponding to the gap and its surroundings (500 bp on both ends), as found by BLASTN (15). A linear contig of 418,588 bp (average coverage, 270×), with a GC content of 33.20%, was generated. GeneMarkS (16) predicted 505 genes using the virus option; 61 of those genes were discarded from the final annotation because they were shorter than 300 bp and had no detectable hits in the nonredundant database (BLASTP E values of <1E−05; pacmanvirus A23 hits were excluded). The 444 predicted open reading frames (ORFs) were functionally annotated according to the best similarities against two protein databases and two motif databases, in the following order: Swiss-Prot and UniRef90 were searched using BLASTP (E values of <1E−05), excluding pacmanvirus A23 hits; Pfam-A and InterPro motif databases were searched using PfamScan (17) and InterProScan (18), respectively (E values of <1E−05). Proteins that did not yield detectable hits were annotated as hypothetical (Fig. 1A). Furthermore, one Ile-tRNA (Fig. 1B) was found by both ARAGON (19) and tRNAscan-SE (20). Pacmanvirus tRNAs were also found in some asfarvirus MAGs (11). For comparison, pacmanvirus A23 was reported to have a smaller genome (395,405 bp) containing 465 predicted protein genes and an Ile-tRNA gene, with a GC content of 33.62% (1). The average nucleotide identity (ANI) between the two strains, as calculated by OrthoANIu (21), was 84.97%.

### Data availability.

Genome and SRA data have been deposited in GenBank under the accession numbers MZ440852 and SRR15690446, respectively.
